# Editorial: The non-neuronal cholinergic system in the cardiovascular system: its influence on the heart, vasculature, and the central nervous system

**DOI:** 10.3389/fcvm.2024.1523385

**Published:** 2024-12-20

**Authors:** Yoshihiko Kakinuma, Takashi Sonobe, Rajesh Katare

**Affiliations:** ^1^ Department of Bioregulatory Science, Graduate School of Medicine, Nippon Medical School, Tokyo, Japan; ^2^ Department of Physiology, School of Biomedical Sciences, University of Otago, Dunedin, New Zealand

**Keywords:** acetylcholine, non-neuronal cardiac cholinergic system (NNCCS), non-neuronal acetylcholine, cardiovascular system, higher brain functions, blood-brain barrer

**Editorial on the Research Topic**
The non-neuronal cholinergic system in the cardiovascular system: its influence on the heart, vasculature, and the central nervous system

In this special issue of Research Topic, authors focus on the significance of non-neuronal acetylcholine (NNA) in the cardiovascular system under different physiological and pathological conditions and further discuss the several NNA-inducing modalities along with extra-cardiovascular beneficial effects.

Many cell types, including immune cells (macrophages and *T* cells) and bronchial epithelial cells, have been reported to produce acetylcholine (ACh). In this Editorial, the term “a non-neuronal cardiac cholinergic system, NNCCS” is used to describe the NNA system in the heart or cardiomyocytes. It has become gradually known that non-neuronal cells, such as cardiomyocytes and endothelial cells (ECs), synthesize Ach (1–3). Cardiomyocyte-derived ACh also plays a role via nicotinic or muscarinic receptors. The heart is regulated by two sources of ACh: the vagus nerve (VN) and cardiomyocytes. Cardiomyocyte-derived ACh synthesis is positively regulated by extrinsic ACh from the VN of the parasympathetic nervous system (PNS), which suggests a positive feedback system (2).

In contrast, as mentioned by Sonobe and Kakinuma, vascular endothelial cells equipped with the ACh synthesis system are less innervated by the PNS. Therefore, it is speculated that NNA in endothelial cells is modulated by different signals than in cardiomyocytes (4–6). ACh also plays a role in cell proliferation (7). Despite earlier biochemical reports showing the presence of NNA in ECs (4–6), few studies have focused on the functions of NNA in ECs, including its vasodilation effects (8). Due to the lower EC-derived ACh levels, difficult detection of EC-specific phenotypes, and more heterogeneous responses of ECs in a vasculature, a study clarifying the role of EC-derived NNA should be carefully conducted and further novel effects should be clarified.

In the cardiovascular system, the physiological and pathophysiological functions of the NNCCS have been intensively studied (1, 2, 9, 10). To summarize, the NNCCS negatively regulates oxygen consumption through the increased glucose utilization by cardiomyocytes, suppresses cardiac mitochondrial dysfunction, and protects cardiomyocytes from ischemia/hypoxia insults, exposure to reactive oxygen species (ROS), and cardiac hypertrophy (2, 9, 10–13). Second, the NNCCS regulates angiogenesis through a non-hypoxic induction pathway involving HIF-1α/VEGF (14). Third, the NNCCS regulates the electrical properties of cardiomyocytes by sustaining the functions of gap junctions (11). Fourth, the NNCCS regulates cardiac responses to an activated sympathetic nervous system (9, 10, 12, 13).

In contrast, the NNCCS influences not only the heart, but also extra-cardiac organs, including the brain, through the VN, as supported by heart-specific ChAT transgenic (hChAT tg) and knockdown mice studies (14–17). NNCCS-activated mice showed increased neuronal activity in the VN through elevated cardiac nitric oxide production, influencing the integrity of the blood-brain barrier (BBB) and higher brain functions. The mice showed unique phenotypes, such as augmentation of anti-anxiety, anti-stress, and anti-depressive phenotypes (15). Even under pathological conditions, the BBB disruption, reactive astrocyte hypertrophy, and proinflammatory cytokine levels were attenuated in the brain (16). Moreover, unlike hChAT tg, vesicular ACh transporter (VAChT) tg mice directly influenced the PNS to further decrease the baseline HR; therefore, responses to a muscarinic receptor antagonist were exaggerated (13).

NNCCS was shown to be downregulated during aging in mice (1). If this is true in humans, the NNCCS may be related to an anti-aging pathway. However, a biomarker that precisely reflects cardiac ACh levels has not been found. As mentioned by Saw et al., a murine diabetic model, db/db mice, showed decreased NNCCS activity during the progression of diabetes mellitus (DM). This was compatible with patients with DM cardiomyopathy (18). In that case, augmentation of the NNCCS may beneficially regulate homeostasis in the heart and the function of the brain. In contrast, ACh levels in patients with non-DM heart failure were not downregulated compared with those in age-matched controls (13). However, it remains to be elucidated whether the NNCCS is finally downregulated or not during heart failure.

Based on these findings, another stage in the development of a therapeutic modality for NNCCS may ensue. As expected, the activation of the NNCCS through pharmacological intervention may be beneficial in humans. In this regard, patients treated with donepezil, an acetylcholinesterase inhibitor often prescribed for Alzheimer's disease, were found to be less susceptible to cardiovascular diseases, and mortality was decreased (19, 20). However, it has already been reported that donepezil upregulates the NNCCS to elevate cardiac ACh levels (2), suggesting that donepezil is an NNCCS inducer. Moreover, recently, S-nitroso-N-pivaloyl-D-penicillamine (SNPiP) has been reported to activate the NNCCS (21), which upregulates cardiac diastolic function (22). In the heart, SNPiP modulates energy metabolism, upregulates hypoxia-responsive pathways with an elevated expression of glycolysis-related enzymes, calcium-handling proteins and atrial natriuretic peptide (ANP), demonstrating that SNPiP positively regulates the NNCCS and enhances calcium handling for efficient cardiac function (23). Remote ischemic preconditioning (RIP) is another modality for enhancing NNCCS, as summarized by Kurabayashi et al. RIP is a well-known modality that exerts anti-ischemic effects on remote organs and it has recently been reported to enhance not only the PNS but also the NNCCS (24). RIP modulates not only cardiac functions but also the functions of extra-cardiac organs, including the liver, as reported by Kurabayashi et al., where glucose metabolism is enhanced to accelerate glucose utilization from the blood (25). As the NNCCS is negatively influenced by glucose intolerance, as reported by Saw et al., RIP itself is expected to be one of the modalities inducing the NNCCS.

A pioneering study (26) reported that the sympathetic nervous system causes pro-inflammatory responses, leading to hypertension, while the cholinergic system ameliorates hypertension (27). Moreover, the anti-inflammatory response by immunocompetent cells, such as macrophages and T cells (28), is expected to influence the progression of cardiovascular diseases.

Therefore, a therapeutic modality that induces NNA or NNCCS is expected to be novel and beneficial for the cardiovascular system, as it is not classified in a conventional category. Further development is expected to provide more specific and effective inducers of this system as adjuvant therapies for cardiovascular diseases.
